# Transcriptomic and Physiological Responses Reveal a Time-Associated Multi-Organ Injury Pattern in European Perch (*Perca fluviatilis*) Under Acute Alkaline Stress

**DOI:** 10.3390/ani15243621

**Published:** 2025-12-16

**Authors:** Geng Chen, Yi Liu, Xiaodong Li, Pan Gao, Jianyong Hu, Pengfei Sun, Fangyuan Peng, Peng Chen, Jin Xu

**Affiliations:** 1 Yangtze River Fisheries Research Institute, Chinese Academy of Fishery Sciences, Wuhan 430223, China; chengeng@yfi.ac.cn (G.C.);; 2 College of Fisheries and Life Sciences, Shanghai Ocean University, Shanghai 201306, China; 3 Xinjiang Uygur Autonomous Region Institute of Fishery Sciences, Urumqi 830099, China

**Keywords:** European perch (*Perca fluviatilis*), alkaline stress, organ-specific responses, oxidative stress, transcriptomics

## Abstract

Water alkalinization is a critical global stressor for freshwater fish, yet the systemic patterns of multi-organ responses and injury remain insufficiently understood. This study integrates histopathology, biochemistry, and multi-organ transcriptomics to provide an integrated, time-resolved assessment of stress responses in European perch (*Perca fluviatilis*) exposed to acute alkaline stress (20 mmol/L). The analysis indicated that alkaline stress initially causes structural disturbance of gill tissue (lamellar fusion, necrosis) within 96 h, associated with impaired osmoregulatory functions. This primary dysfunction was followed by progressive hepatic impairment, characterized by uncontrolled oxidative stress (elevated levels in Malondialdehyde, MDA) and widespread hepatocyte necrosis. Transcriptomic analysis identified extensive transcriptional shifts associated with these alterations: large-scale differential expression in the liver (3629 Differentially Expressed Genes, DEGs) and kidney (478 DEGs). Notably, the liver exhibited a stress-responsive transcriptional profile involving activation of the HIF-1 signaling pathway and mobilizing protein quality control systems (e.g., ‘Proteasome,’ ‘Lysosome’) consistent with mitigation of proteotoxic stress. This compensatory response appeared insufficient to prevent severe metabolic disruption and cellular injury. This study presents a time-associated sequence of organ-specific stress responses under acute alkalinity, identifying candidate stress-associated genes (*slc7a11*, *egln3*, *klhl38b*) as potential targets for future functional studies and breeding alkali-tolerant strains.

## Simple Summary

Freshwater lakes and rivers worldwide are becoming increasingly alkaline, presenting a growing challenge for fish health and aquaculture. This study investigated how high-alkaline water affects European perch (*Perca fluviatilis*), an important species for food and healthy ecosystems. We aimed to understand the time-associated sequence of physiological and molecular changes that occur in the fish’s body during acute alkaline stress. Our results showed that alkaline water first induces early structural disturbance in the gills, the organ fish use to breathe and balance their internal salts. This initial disturbance is followed by progressive dysfunction in the liver, accompanied by oxidative imbalance and metabolic disruption. We also identified several stress-related genes showing strong transcriptional responses. This research provides a time-resolved overview of organ-specific stress responses under alkaline exposure. For society, these findings highlight potential genetic candidates that may support future breeding programs aimed at enhancing alkaline tolerance in aquaculture species.

## 1. Introduction

Globally, the alkalinization of freshwater bodies is becoming increasingly prevalent due to industrial effluent discharge, agricultural fertilizer runoff, and natural processes in certain geological regions [1]. Water alkalinization, typically characterized by an elevated pH and a significant increase in carbonate and bicarbonate ion concentrations, poses severe osmotic and acid–base balance challenges to the survival of most freshwater fish [2]. For modern aquaculture, which relies on stable water quality, water alkalinization not only restricts the selection of farming areas but also directly threatens the health and yield of cultured species, becoming a key environmental factor constraining the sustainable development of the industry [2]. The European perch (*Perca fluviatilis*), a widely distributed species with significant ecological and commercial value in Europe, is also an important species for intensive aquaculture [3]. Concurrently, China possesses vast areas of saline–alkali waters, which are difficult to utilize for traditional fisheries due to their high pH and complex ionic composition [4]. Therefore, cultivating new alkali-tolerant fish varieties that can adapt to and utilize these waters holds significant strategic importance for expanding aquaculture space and ensuring food security [5].

High-pH and high-alkalinity environments present multiple physiological challenges for teleost fish, with the most critical being osmoregulation, acid–base balance, and nitrogen metabolism [6]. First, the gills, as the central organ for material exchange between the fish and the aquatic environment, experience severe disruption to their ion regulatory function. Unfavorable ion gradients significantly hinder the active absorption of Na^+^ and Cl^−^ by branchial epithelial cells via ion exchangers (such as Na^+^/H^+^ exchangers), leading to critical ion loss and osmotic imbalance [7]. Second, the combination of high external water pH and high bicarbonate concentration (high alkalinity) impedes the fish’s ability to effectively excrete metabolic waste. High pH reduces the P_CO2_ diffusion gradient between the blood and water, while high alkalinity may reverse Cl^−^/HCO_3_^−^ exchange. Together, these factors cause internal CO_2_ accumulation (hypercapnia) and/or metabolic alkalosis, severely threatening acid–base homeostasis [8]. Finally, a high-pH environment significantly suppresses nitrogenous waste excretion in the form of ammonia via the gills by reducing the NH_3_ diffusion gradient and inhibiting NH_4_^+^ exchange mechanisms. This leads to the accumulation of ammonia in the blood and tissues (hyperammonemia) and potent neurotoxicity, which is a primary cause of acute mortality in fish in high-pH/high-alkalinity waters [9].

At the cellular level, oxidative stress is a universal damage mechanism employed by organisms subjected to various environmental stimuli, including extreme pH [10]. Environmental stress disrupts the balance between the production and scavenging of Reactive Oxygen Species (ROS) within cells, leading to their excessive accumulation [11]. Excess ROS attack biomacromolecules such as cell membranes, proteins, and nucleic acids through lipid peroxidation, protein carbonylation, and DNA damage, causing functional impairment [12]. Although organisms possess a sophisticated antioxidant defense system—the first line of which includes enzymes like Superoxide Dismutase (SOD), Catalase (CAT), and Glutathione Peroxidase (GSH-Px) [13]—this system can be overwhelmed by severe or persistent stress, ultimately leading to oxidative damage [10].

To move beyond phenomenological descriptions and probe the molecular mechanisms of stress response, transcriptomic technology (RNA-Seq) provides a powerful tool [14]. By measuring gene expression levels across the entire genome, RNA-Seq can reveal key genes and signaling pathways activated or suppressed under specific stress conditions. In recent years, this technology has been widely applied to study fish responses to high-pH and high-alkalinity stress. For instance, transcriptomic analysis of silver crucian carp (*Carassius gibelio*) under long-term high-alkalinity stress revealed significant changes in genes related to metabolic and immune pathways in the gills and kidneys [15]. Similarly, transcriptomic analysis of gill, liver, and kidney tissues from grass carp (*Ctenopharyngodon idella*) under alkali stress also identified numerous associated differentially expressed genes and pathways [16]. Thus, RNA-Seq technology helps construct molecular networks of stress response and fundamentally understand the molecular basis of injury and adaptation across different time scales.

While previous studies have documented organ-specific damage, a time-resolved description of how different organs respond sequentially to alkaline exposure in *P. fluviatilis* remains elusive. This gap prevents a more comprehensive understanding of acute alkaline toxicity. Therefore, the core objectives of this study were (1) to characterize the temporal evolution of histopathological damage in the primary target organs (gills and liver) through histological observation; (2) to quantify the dynamic biochemical response, particularly hepatic oxidative stress, via biochemical assays; (3) to utilize multi-organ transcriptomics to identify transcriptomic signatures associated with stress responses in the liver and kidney; and (4) to integrate these multi-level data to provide an integrated view of organ-specific and temporal stress responses to acute alkaline stress, supporting future investigation into alkaline stress mechanisms and for informing potential breeding research on alkaline tolerance.

## 2. Materials and Methods

### 2.1. Experimental Animals and Acclimation

European perch were obtained from the Xinjiang Aquatic Wild Animal Rescue Center. The experiment used healthy juvenile *Perca fluviatilis* with an average body length of 15.98 ± 1.12 cm and weight of 40.14 ± 12.86 g. Due to the juvenile stage, sex was not differentiated, but individuals were randomly assigned to groups to minimize potential bias. During the acclimation and experimental phases, dissolved oxygen was maintained above 6 mg/L, ammonia-nitrogen and nitrite-nitrogen levels were kept below 0.01 mg/L, and water temperature was held at 22 °C. Fish were acclimated to these conditions for one week. To ensure experimental accuracy, all fish were fasted for 24 h before the trial and were not fed during the experiment.

### 2.2. Alkaline Stress Experimental Design

**Figure 1:**
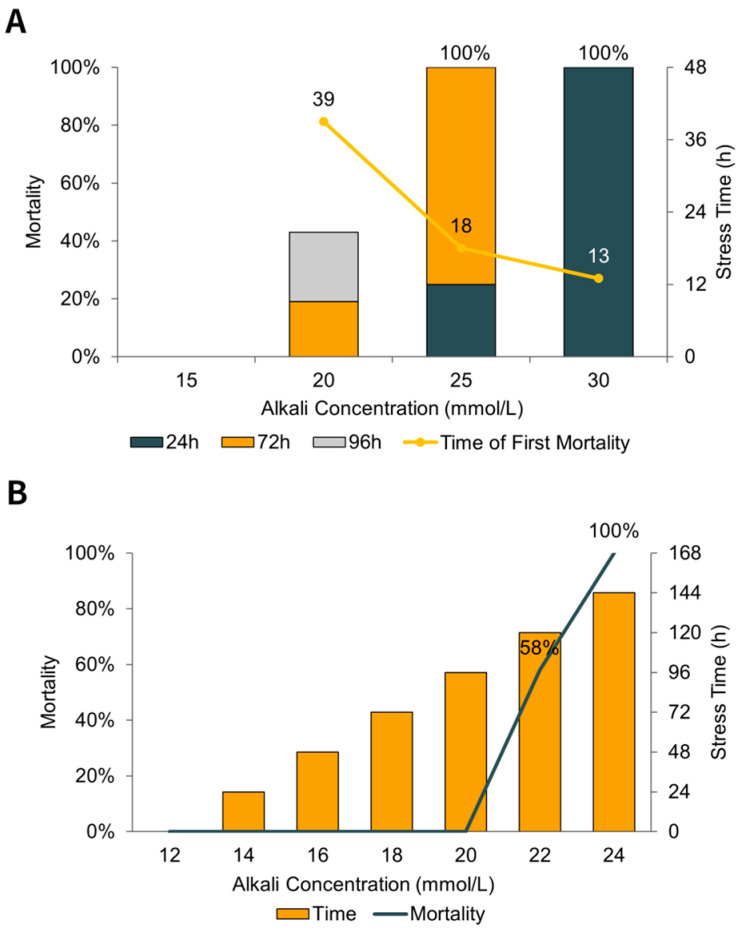
Mortality of *P. fluviatilis* under alkaline stress. (**A**) Mortality rates at 24, 72, and 96 h under different fixed alkalinity concentrations (15, 20, 25, 30 mmol/L). The yellow line indicates the time of the first death at each concentration. (**B**) Cumulative mortality rate under progressively increasing alkalinity stress.

To determine the median lethal concentration (LC50), preliminary experiments were conducted testing the 96-h mortality rate of perch at different fixed alkali concentrations (10–30 mmol/L) and gradually increasing concentrations. The detailed results of these preliminary tests are presented in Figure 1. Based on these mortality curves and tolerance thresholds (Figure 1), a concentration of 20 mmol/L was selected for the acute stress experiment. The alkaline stress solution (20 mmol/L) was prepared using a buffer mixture of sodium bicarbonate (NaHCO_3_) and sodium carbonate (Na_2_CO_3_) added to dechlorinated tap water. A specific mass ratio of 16.1:1 (NaHCO_3_:Na_2_CO_3_) was utilized to stabilize the pH at 9.4 ± 0.3. Based on stoichiometry, this buffer system introduced approximately 20.0 mmol/L of exogenous sodium ions (Na^+^). Water quality parameters, including pH, dissolved oxygen (DO), temperature, and conductivity, were measured using a YSI ProPlus multiparameter meter (YSI Inc., Yellow Springs, OH, USA). Total alkalinity and water hardness were determined by acid–base titration and EDTA titration, respectively, while ammonia-nitrogen (TAN) was quantified using Nessler’s colorimetry. Major ions (Na^+^, K^+^, Ca^2+^, Mg^2+^) were measured using flame photometry or EDTA titration, depending on ion type. All analyses followed standard aquatic toxicology procedures to ensure experimental reliability. The detailed calculated and measured water chemistry parameters are listed in Table 1.

The experiment was conducted in 250 L circular plastic tanks. The experimental solution was prepared and allowed to settle overnight. Ninety perch of uniform size and free of injury were randomly distributed into the tanks, with 30 fish per tank and three replicate tanks per group. Water temperature was maintained at 22 °C using a PTC variable-frequency heater (Chuangning, Shanghai, China). The acute stress test lasted 96 h. Continuous aeration was provided to maintain dissolved oxygen above 5 mg/L. To maintain stable alkalinity and remove metabolic waste, one-third of the solution was replaced every 24 h with a new solution prepared in advance to match the original pH and alkalinity.

### 2.3. Sample Collection and Processing

Liver, kidney, and gill tissues were collected at six time points (0, 12, 24, 48, 72, and 96 h). At each point, three fish were randomly sampled from each replicate, anesthetized with MS-222 (0.03 g/L), and their length and weight were measured. Tissues were collected under sterile, low-temperature conditions. Samples for enzyme activity assays were placed in cryovials and immediately stored at −80 °C. Samples for gene expression analysis were placed in RNAlater (Thermo Fisher Scientific, Waltham, MA, USA), stored at 4 °C for 24 h, then the solution was discarded, and samples were stored at −80 °C. Samples for histology were fixed in 4% paraformaldehyde and stored at 4 °C. Three independent biological replicates (*n* = 3 individual fish) were sequenced for each group.

### 2.4. Histological and Biochemical Analysis

Gill and liver tissue samples were fixed in 4% paraformaldehyde for 24 h. Tissues were then processed using standard histological procedures: dehydrated through a graded ethanol series, cleared in xylene, embedded in paraffin, and sectioned. Sections were stained with Hematoxylin-Eosin (H&E) and mounted with neutral resin. Images were captured using an optical microscope (Nikon ECLIPSE Ts2, Shinagawa, Japan) for analysis.

Antioxidant enzyme activities, including Catalase (CAT), Superoxide Dismutase (SOD), and Glutathione Peroxidase (GSH-Px), as well as Malondialdehyde (MDA) content, were measured in liver homogenates using commercial kits (Nanjing Jiancheng Bioengineering Institute, Nanjing, China). Absorbance was read using a spectrophotometer (MAPADA UV1100, Shanghai, China). Protein concentration in the homogenates was determined using the BCA method.

### 2.5. RNA Extraction, Transcriptome Sequencing, and Bioinformatics Analysis

Total RNA was extracted from homogenized perch tissues using the TRIzol method and stored at −80 °C [17]. RNA purity and concentration were assessed using an Implen micro-volume spectrophotometer and agarose gel electrophoresis. Samples meeting the quality criteria (Total RNA > 2 µg; OD260/280 ratio 1.9–2.1; concentration > 300 ng/µL) were used for sequencing. Library construction and sequencing were performed by Guangzhou Gidio Co., Ltd. (Guangzhou, China) using the Illumina NovaSeq 6000 platform.

To ensure data quality, raw reads were first processed using fastp (version 0.20.0) to obtain clean reads by (1) removing adapter-containing reads, (2) removing reads with >10% N content, (3) removing reads composed solely of ‘A’ bases, and (4) removing low-quality reads (where >50% of bases had Q ≤ 20) [18]. High-quality clean reads were then aligned to the *P. fluviatilis* reference genome using HISAT2 (v2.2.1) [19,20]. Gene expression levels were quantified as FPKM (Fragments Per Kilobase per Million bases) using RSEM (RNA-Seq by Expectation-Maximization) [21,22]. Differentially expressed genes (DEGs) between control and stress groups were identified using DESeq2 (v1.42.0) [23]. The thresholds for significant differential expressions were set at a False Discovery Rate (FDR) < 0.05 and an absolute |log2(FoldChange)| > 1.

DEGs were subjected to Gene Ontology (GO) functional annotation and Kyoto Encyclopedia of Genes and Genomes (KEGG) pathway enrichment analysis [24,25]. Enrichment analysis was performed using the hypergeometric distribution method in R (v4.3.3), with the Benjamini–Hochberg (BH) method applied for multiple testing correction [26]. GO terms and KEGG pathways with an FDR ≤ 0.05 were considered significantly enriched.

### 2.6. cDNA Synthesis and qRT-PCR Validation

Total RNA was extracted from liver tissue using the TRIzol method. After quality assessment, 1000 ng of total RNA was reverse transcribed into cDNA using the PrimeScript RT Reagent Kit (TaKaRa, Kusatsu, Japan) according to the manufacturer’s protocol: 25 °C for 10 min, 55 °C for 50 min, and 85 °C for 5 min. Synthesized cDNA was stored at −20 °C.

To validate the RNA-Seq data, eight representative DEGs were selected for quantitative real-time PCR (qRT-PCR) analysis using three independent biological replicates. The qRT-PCR was performed using a QuantStudio 1 Plus Real-Time PCR System (Applied Biosystems, Thermo Fisher Scientific). Each 10 μL reaction contained 1 μL of cDNA template derived from 1000 ng of total RNA used for reverse transcription. qRT-PCR was performed using TB Green^®^ Premix Ex TaqTM (TaKaRa) with the following reaction system: 5 µL 2× S6 Universal SYBR qPCR mix, 0.2 µL each of forward and reverse primers, 1 µL cDNA, and 3.6 µL ddH2O. The thermal profile was 95 °C for 30 s; 40 cycles of 95 °C for 5 s and 60 °C for 34 s; followed by a melt curve analysis. Relative gene expression levels were calculated using the 2^−ΔΔCT^ method [27]. All primer sequences (Supplementary Material Table S1) were synthesized by Tianyi Huayu Co., Ltd. (Wuhan, China).

### 2.7. Statistical Analysis

Experimental data were expressed as mean ± standard deviation (Mean ± SD). One-way analysis of variance (ANOVA) was performed using IBM SPSS Statistics 27. The significance level was set at *p* < 0.05.

## 3. Results

### 3.1. Alkaline Stress Experiment

During the experiment, fish were fasted to prevent metabolic interference; thus, feeding activity was not assessed. However, significant behavioral stress responses were observed in the alkali-treated groups prior to mortality. Affected fish exhibited increased respiratory frequency (rapid opercular movement) and erratic swimming, followed by a loss of equilibrium (LOE). Mortality was confirmed by the complete cessation of opercular movement. In the 15 mmol/L alkali stress group, no perch mortalities were observed. In the 20 mmol/L group, the first mortality occurred at 39 h; the mortality rate was 19% at 72 h and 43% at 96 h. In the 25 mmol/L group, mortality occurred as early as 18 h, with a 25% mortality rate at 24 h and 100% at 72 h. In the 30 mmol/L group, mortality began at 13 h and reached 100% by 24 h (Figure 1A). In a separate trial with progressively increasing alkalinity, no mortality occurred up to 20 mmol/L (96 h). However, when the concentration increased to 22 mmol/L (120 h), mortality reached 58%, and at 24 mmol/L, all fish perished (Figure 1B).

### 3.2. Acute Alkaline Stress Induces Rapid and Progressive Pathological Damage in Gills and Liver

Based on the mortality results, 20 mmol/L was selected as the concentration for time-series analysis, as it induced significant sublethal and lethal effects within 96 h, allowing for the study of dynamic injury processes.

#### 3.2.1. Structural Degeneration of Gill Tissue

**Figure 2:**
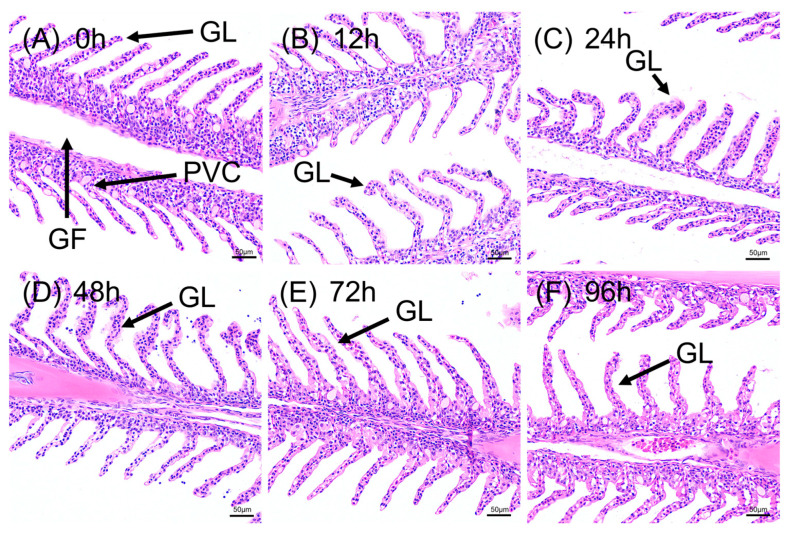
Histopathological changes in the gills of *P. fluviatilis* under acute alkaline stress (H&E staining). (**A**) Control group (0 h) showing normal gill filaments (GF) and gill lamellae (GL). (**B**–**F**) Progressive damage at 12, 24, 48, 72, and 96 h, characterized by edema, lamellar bending, epithelial lifting, and necrosis. PVC: pavement cells. Scale bar = 50 μm.

Acute alkaline stress caused increasingly severe structural alterations to the gill over 96 h (Figure 2). Control (0 h) gills showed intact, neatly arranged Gill Filaments (GF) and Gill Lamellae (GL). After 12 h of stress, edema and swelling at the lamellar tips were observed. By 24 h, the edema intensified, causing lamellar bending and deformation. At 48 h, tissue damage became more pronounced, with widespread lamellar fusion and epithelial lifting. In the final phase (72–96 h), the gill architecture appeared severely disrupted, with extensive epithelial lifting and areas of cell necrosis, suggesting substantial impairment of respiratory and osmoregulatory capacity.

#### 3.2.2. Pathological Changes in Liver Tissue

**Figure 3:**
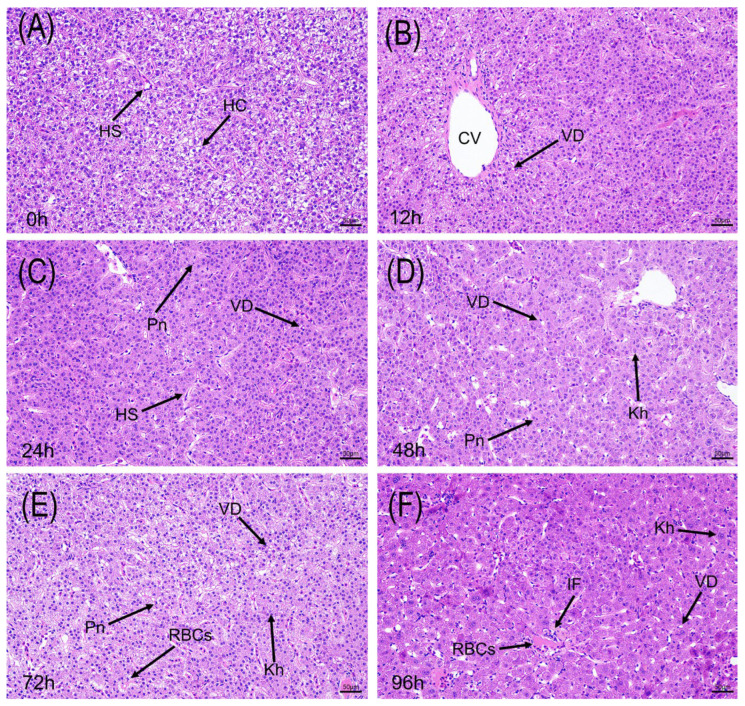
Histopathological changes in the liver of *P. fluviatilis* under acute alkaline stress (H&E staining). (**A**) Control group (0 h) showing normal hepatocytes (HC) and hepatic sinusoids (HS). (**B**–**F**) Progressive damage at 12, 24, 48, 72, and 96 h, characterized by vacuolar degeneration (VD; formation of cytoplasmic vacuoles), pyknosis (Pn; condensed and dark nuclei), karyorrhexis (Kh; nuclear fragmentation), inflammatory cell infiltration (IF), and red blood cell congestion (RBCs). CV: central vein. Scale bar = 50 μm.

The liver, as the central metabolic and detoxification organ, showed progressively more apparent structural and cellular alterations under alkaline stress (Figure 3). Control (0 h) hepatocytes (HC) were polygonal with large, round nuclei and clear cell boundaries. After only 12 h of stress, edema and vacuolar degeneration (VD, appearing as cytoplasmic empty vacuoles) were observed in some hepatocytes. By 24 h, this damage became more prominent, with enlarged hepatic sinusoids (HS) due to tissue swelling, and more prevalent vacuolization. At 48 h, the damage was characterized by extensive hepatocyte injury, marked by widespread hepatocyte necrosis. This was evidenced by nuclear pyknosis (Pn), where nuclei became shrunken and hyperchromatic, and karyorrhexis (Kh), characterized by the fragmentation of nuclei into scattered, distinct bodies. In the final phase (72–96 h), the liver structure appeared substantially disrupted, accompanied by inflammatory cell infiltration (IF) and congestion of red blood cells (RBCs), suggesting marked impairment of hepatic function under acute alkaline exposure.

### 3.3. Hepatic Oxidative Stress Response

**Figure 4:**
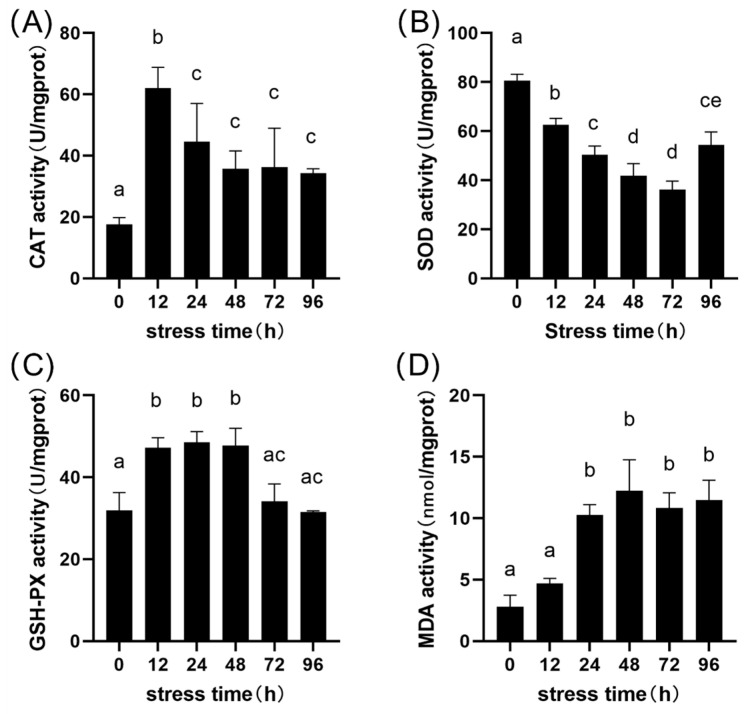
Effects of acute alkaline stress on the antioxidant enzyme activities in the liver of *P. fluviatilis*. Activities of (**A**) Catalase (CAT), (**B**) Superoxide dismutase (SOD), (**C**) Glutathione peroxidase (GSH-Px), and (**D**) content of Malondialdehyde (MDA) were measured at 0, 12, 24, 48, 72, and 96 h post-stress. Data are presented as mean ± standard deviation (SD, *n* = 3). Different lowercase letters (a–e) above the bars indicate significant differences between time points (*p* < 0.05). Bars sharing the same letter are not significantly different.

To investigate the biochemical mechanisms underlying the tissue damage, key oxidative stress indicators were measured in liver homogenates. As shown in Figure 4, the antioxidant response was dynamic. CAT and GSH-Px activities both showed a significant increase followed by a decrease, peaking at 12 h and 24 h, respectively (*p* < 0.05). Conversely, SOD activity significantly decreased initially before recovering. In stark contrast, the lipid peroxidation marker, MDA content, showed a continuous and significant increasing trend throughout the stress period, plateauing at an extremely high level after 48 h (*p* < 0.05).

### 3.4. Transcriptome Sequencing Reveals Large-Scale Gene Reprogramming in Liver and Kidney

To analyze the molecular response to alkaline stress, samples from the 24 h time point were selected for RNA-Seq. This point represents the critical transition from initial stress to significant organ damage. A total of 12 samples (liver control, liver stress, kidney control, kidney stress; *n* = 3 per group) were sequenced. After quality control, a total of 77.99 Gb of high-quality clean data was obtained. Each sample yielded an average of 43.50 ± 4.94 million clean reads. All samples demonstrated excellent quality, with Q20 scores ranging from 98.2% to 98.82%, Q30 scores from 94.86% to 96.5%, and stable GC content (47.61% to 50.73%), as detailed in Supplementary Material Table S2. Hierarchical clustering analysis demonstrated that samples were primarily grouped by tissue type (kidney vs. liver), followed by treatment conditions (control vs. alkali stress). High correlations within biological replicates confirmed the reproducibility of the data (Supplementary Material Figure S1).

**Figure 5:**
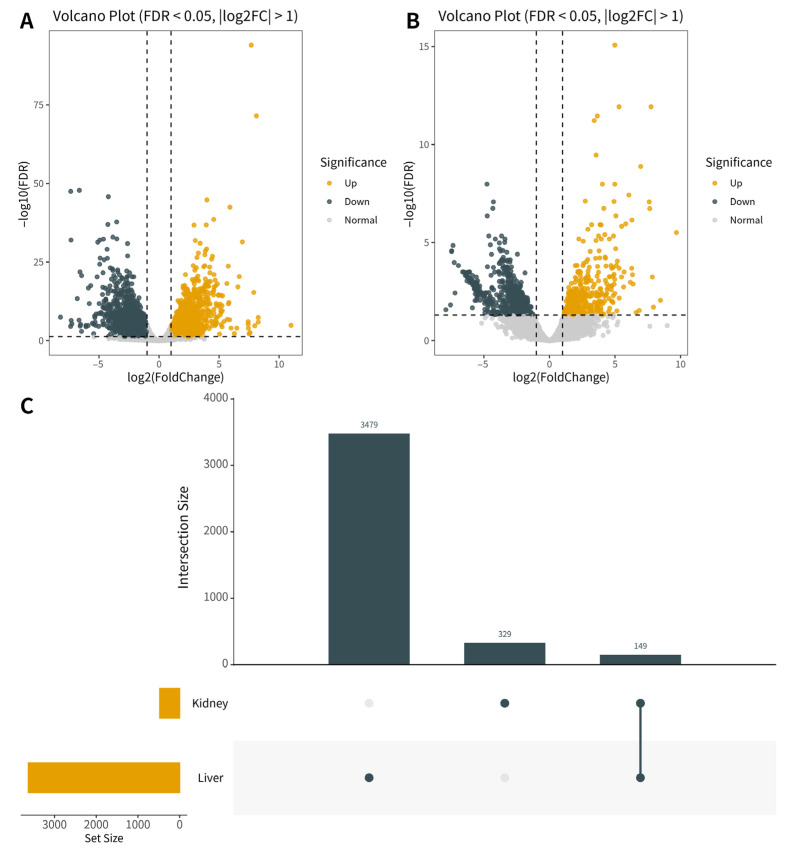
Analysis of differentially expressed genes (DEGs) in *P. fluviatilis* under acute alkaline stress. (**A**,**B**) Volcano plots showing the distribution of DEGs in the (**A**) liver and (**B**) kidney. Red dots represent upregulated genes, green dots represent down-regulated genes, and blue dots represent non-significantly expressed genes. The vertical and horizontal dashed lines indicate the thresholds of |log2(FoldChange)| = 1 and FDR = 0.05, respectively. (**C**) UpSet plot visualizing the intersection of DEGs between the liver and kidney. The vertical bars represent the number of unique or shared genes, while the connected dots below indicate the specific intersection sets.

In the liver, 3629 DEGs were identified (1966 upregulated, 1663 downregulated) (Figure 5A). In the kidney, 478 DEGs were identified (196 upregulated, 282 downregulated) (Figure 5B). Although the number of DEGs in the kidney was much smaller than in the liver, it still indicates its involvement in the stress response, potentially in a more specific or secondary capacity.

To further elucidate the tissue-specific and conserved molecular responses, we analyzed the overlap of DEGs between the two organs using an UpSet plot (Figure 5C). A total of 149 genes were co-expressed in both tissues, suggesting a core set of genes involved in the systemic response to alkaline stress. In contrast, 3479 and 329 genes were uniquely regulated in the liver and kidney, respectively. Functional characterization of these 149 conserved DEGs revealed these genes were enriched in energy and lipid metabolism (e.g., *pfkfb3*, *ldha*, *dgat2*), stress response (e.g., *hsp90aa1.2*, *cat*) and acid–base regulation (e.g., *atp6v1ab*, *tcirg1b*). Detailed information for all differentially expressed genes can be found in the Supplementary Material S2.

### 3.5. Functional Enrichment Analysis of DEGs

To understand the biological significance of these transcriptional changes, GO and KEGG analyses were performed.

In the liver, DEGs were significantly enriched in GO terms such as “Cellular process,” “Metabolic process,” and “Biological regulation” (Biological Process); “binding” and “catalytic activity” (Molecular Function); and “cellular anatomical entity” and “protein-containing complex” (Cellular Component) (Supplementary Material S1 Figure S2).

In the kidney, DEGs were similarly enriched in “cellular process,” “metabolic process,” and “response to stimulus” (Biological Process); “binding” and “catalytic activity” (Molecular Function); and “cellular anatomical entity” and “protein-containing complex” (Cellular Component) (Supplementary Material S1 Figure S4).

KEGG pathway analysis provided deeper insight. In the liver, DEGs were significantly enriched in “Metabolic pathways,” “Proteasome,” “Carbon metabolism,” and “Spliceosome,” suggesting that the liver’s response involved major shifts in energy metabolism, protein degradation, and RNA processing (Supplementary Material S1 Figure S3).

In contrast, kidney DEGs were primarily enriched in “Metabolic pathways,” “Lysosome,” “Glyoxylate and dicarboxylate metabolism,” and “Regulation of lipolysis in adipocytes,” indicating a response focused on catabolism, lysosomal activity, and specific energy pathways (Supplementary Material S1 Figure S5).

### 3.6. Key Differentially Expressed Genes

To identify core regulatory genes, a subset of the most significantly up- and down-regulated genes are listed in Table 2. In the kidney, the immune-related gene *acod1* and the ion transport-related *slc7a11* were strongly upregulated, while metabolic genes like *uox* and *fabp10a* were downregulated. In the liver, *stard5*, *aldh18a1*, *higd1a*, *egln3*, and *slc7a11* were strongly upregulated, while genes like *angptl3* and *uox* were strongly downregulated. These genes represent important candidates for understanding the stress mechanism and for future alkali-tolerant breeding.

### 3.7. Validation of RNA-Seq Data by qRT-PCR

**Figure 6:**
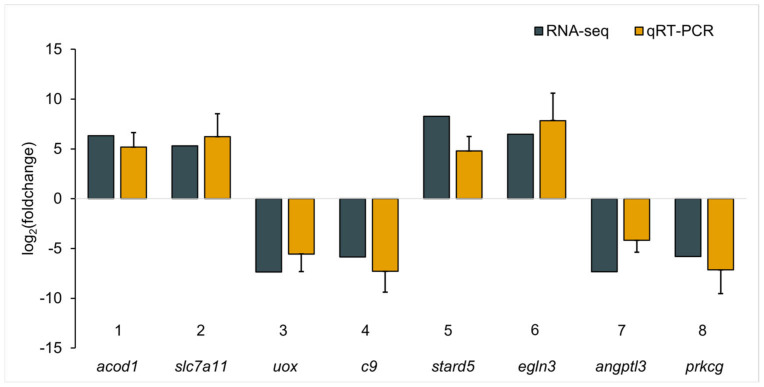
Validation of RNA-Seq data by qRT-PCR. Comparison of gene expression levels obtained from RNA-Seq (log2(FoldChange)) and qRT-PCR (Relative Expression) for eight representative DEGs. (1–4) Four genes selected from kidney tissue. (5–8) Four genes selected from liver tissue. The qRT-PCR data are presented as mean ± SD (*n* = 3).

To validate the accuracy and reliability of the RNA-Seq data, eight representative DEGs (four from kidney, four from liver) were selected for qRT-PCR analysis. As shown in Figure 6, the relative expression trends (up- or downregulation) measured by qRT-PCR were highly consistent with the log2(FoldChange) values from the RNA-Seq data. This high correlation confirms the reliability of the transcriptomic results and provides a solid foundation for the functional analysis.

## 4. Discussion

The histopathological results indicate that the gills and liver as the two primary organs damaged by acute alkaline stress, which aligns with observations in other freshwater fish [28,29]. The gills, as the primary interface with the environment, are the first-line defense and thus the first to be injured [6]. In high pH water, the ammonia equilibrium shifts toward the more toxic non-ionic ammonia (NH3), which can enhance the toxic effects on gill tissues [30]. The observed lamellar swelling, fusion, and epithelial lifting are consistent with typical pathological features of high-alkalinity or ammonia toxicity. These alterations disrupt the gill architecture and are associated with impaired ion regulation and gas exchange, which in turn rapidly induces systemic physiological disorders [31].

The hepatic alterations occur later in the exposure period, coinciding with increased oxidative stress indicators, suggesting a delayed and possibly stress-linked response. The hepatic necrosis observed after 48 h (Figure 3) is accompanied by biochemical signs of oxidative imbalance. The biochemical data show that the initial compensatory spikes in CAT and GSH-Px (Figure 4A,C) were insufficient to counter the ROS influx, a pattern of antioxidant imbalance also seen in other alkali-stressed fish [32]. The antioxidant defense system was overwhelmed, resulting in the uncontrolled, continuous rise in the lipid peroxidation marker MDA (Figure 4D). This indicates catastrophic cell membrane destruction and aligns perfectly with the timing of the irreversible histological collapse (necrosis, pyknosis) observed at 48 h (Figure 3D) [33]. Overall, the time-course observations outline a sequence of organ-specific stress responses under alkaline exposure rather than a fully verified cascade of injury.

The 24 h transcriptome provides a molecular snapshot of this crisis, revealing the complex strategies employed by the cells to survive. The liver’s transcriptional response reflects a large-scale energy reallocation, with enrichment of ‘Carbon metabolism’ and ‘TCA cycle’ pathways, representing the mobilization of energy reserves to fuel defense mechanisms [34]. More critically, the cell activated massive protein quality control (PQC) systems to cope with the damage. The significant enrichment of the ‘Proteasome’ pathway in the liver is direct evidence of a cellular attempt to clear a flood of damaged, misfolded proteins generated by oxidative stress [35]. This is supported at the gene level by the strong upregulation of *klhl38b* and *asb10* (Table 2), which are key components of the E3 ubiquitin ligase complex that targets proteins for degradation [36,37,38]. The ‘Spliceosome’ enrichment further suggests a sophisticated response, possibly activating alternative splicing to rapidly diversify protein function from single genes to meet the complex stress challenge [39].

In contrast, the kidney’s more targeted response centered on the ‘Lysosome’ pathway. This suggests a supportive role as a systemic ‘filtration and processing center,’ activating autophagy and phagocytosis to clear circulating cellular debris and toxins generated by the primary gill and liver failure [40]. The enrichment of pathways like ‘Glyoxylate and dicarboxylate metabolism’ and ‘Alanine, aspartate and glutamate metabolism’ in the kidney also points to its specific role in managing acid–base balance and metabolic waste [41].

Analysis of key DEGs reveals a sophisticated but ultimately futile defense strategy. The transcriptome shows a coordinated effort to synthesize new glutathione (GSH), a key antioxidant, via the strong upregulation of *slc7a11* (xCT), which mediates the rate-limiting step of cystine uptake [42]. Simultaneously, the cell attempted to preserve the potent endogenous antioxidant uric acid via the strong downregulation of *uox* (urate oxidase), the enzyme that degrades it [43]. The failure of this multi-pronged strategy, as evidenced by the soaring MDA levels (Figure 4D), underscores the extreme severity of stress.

A critical finding is the paradoxical regulation of the HIF-1 signaling pathway. Both the HIF-1 target gene *higd1a* (which remodels mitochondria) and the key HIF-1 inhibitor *egln3* (which promotes HIF-1 degradation) were strongly upregulated (Table 1). This contradictory signaling suggests a complete breakdown of homeostatic feedback. The cell, likely sensing hypoxia or ROS, activates HIF-1 (driving *higd1a*), but this activation appears dysfunctional, triggering a simultaneous, massive, and futile upregulation of its own inhibitor (*egln3*), highlighting a catastrophic loss of regulatory control [44,45]. Other key genes, such as *prlra* (prolactin receptor), point to the involvement of high-level endocrine control of osmoregulation, while *aldh18a1* suggests synthesis of proline as an osmoprotectant [46,47]. The shifts in *fabp10a* and *angptl3* indicate a total reprogramming of lipid metabolism, likely to fuel the massive energy demand, and *ucp1* activation suggests mitochondrial uncoupling [48,49,50]. Finally, the downregulation of *c9* (Complement component 9) may be a signal of complex immune and inflammatory modulation [51].

The biochemical indicators revealed a progressive imbalance in oxidative status under alkaline exposure, whereas the transcriptomic snapshot at 24 h showed mixed regulation of classical antioxidant enzymes. This apparent inconsistency can be interpreted by considering the different biological scales captured by each dataset. Malondialdehyde (MDA) accumulates as a cumulative outcome of membrane lipid peroxidation, whereas antioxidant enzyme activities (CAT, SOD, GSH-Px) may fluctuate rapidly due to substrate availability and post-translational modification. In contrast, RNA-Seq reflects an instantaneous transcriptional state that may precede or deviate from biochemical measurements during acute stress.

Notably, we observed strong upregulation of the cystine/glutamate antiporter slc7a11, which facilitates glutathione (GSH) biosynthesis, while GSH-Px activity declined at later stages. Similar asynchronous patterns have been reported in aquatic animals under environmental stress, in which transcriptional responses can precede measurable biochemical compensation, or enzyme activities may decline despite transcriptional support under overwhelming oxidative burden [10,11]. Moreover, enrichment of proteasome- and lysosome-related genes (e.g., klhl38b, asb10) suggests that protein quality control (PQC) mechanisms could contribute to early stress management by reducing ROS-induced protein misfolding prior to potential enzyme exhaustion [40].

Therefore, the combined observations likely represent a non-synchronous but complementary process, in which early transcriptional activation may support precursor metabolism and protein degradation systems, while biochemical indicators reflect delayed oxidative outcomes once reactive oxygen species exceed cellular buffering capacity. Thus, the divergence between biochemical and transcriptomic patterns is better interpreted as a temporal difference in oxidative stress regulation rather than inconsistency between datasets.

Although kidney histology was not examined in the present study, transcriptomic data showed shifts in several functional pathways. These transcriptional changes may reflect early molecular adjustments potentially related to metabolic and lysosome-associated processes during alkaline exposure. Given the absence of histopathological validation, we do not infer structural injury in the kidney and interpret these signatures only as indicators of possible functional modulation. Future work including kidney morphology will be necessary to determine whether these molecular responses correspond to anatomical alterations.

While this study offers a time-resolved description of acute alkaline stress responses, its limitations must be acknowledged. First, the acute (96 h) high-concentration stress simulates a sudden pollution event but may not fully represent the chronic, low-level exposure common in aquaculture. Future studies should explore adaptive responses under chronic stress. Second, although the transcriptomic findings suggest involvement of HIF-1 and PQC pathways, further work is needed to determine whether these transcriptomic signals correspond to protein-level or metabolic adjustments. Finally, the protective roles of candidate genes (*slc7a11*, *egln3*, *klhl38b*) are currently hypothesized, and functional studies (e.g., gene knockout/overexpression) could help clarify whether they contribute to alkaline tolerance. Such work may support future investigations into molecular selection strategies, rather than directly providing breeding markers at this stage.

## 5. Conclusions

In conclusion, this study provides a time-resolved overview of the physiological and molecular responses of *Perca fluviatilis* to acute alkaline stress. The findings outline a sequence in which gill tissues are affected earlier, followed by pronounced hepatic changes, in association with oxidative imbalance during prolonged exposure. Transcriptomic signals related to antioxidant processes and protein quality control (e.g., proteasome, lysosome) suggest potential involvement of stress-response pathways, although functional outcomes remain to be validated. The significance of this work is twofold: it highlights organ-specific vulnerability under acute alkaline exposure, and it identifies stress-associated genes (*slc7a11*, *egln3*, *klhl38b*) as potential candidates for future functional and breeding-related research.

## Figures and Tables

**Table 1:** Physicochemical parameters of the water during the 96-h acute alkaline stress test.

Parameter	Control Group	Alkaline Stress Group	Method
pH	7.8 ± 0.2	9.4 ± 0.3	YSI ProPlus Meter
Total Alkalinity (mmol/L)	3.2 ± 0.3	20.0 ± 0.2	Acid–Base Titration
Total Hardness (mg/L CaCO_3_)	180.5 ± 4.2	168.7 ± 6.5	EDTA Titration
Dissolved Oxygen (mg/L)	7.6 ± 0.4	7.4 ± 0.6	YSI ProPlus Meter
TAN (mg/L)	<0.02	<0.02	Nessler’s Colorimetry
Ca^2+^ (mg/L)	54.1 ± 2.8	49.5 ± 3.2	EDTA Titration
Mg^2+^ (mg/L)	10.9 ± 1.5	10.8 ± 1.8	Calculated
Na^+^ (mg/L)	32.5 ± 3.4	482.2 ± 10.6	Flame Photometry
K^+^ (mg/L)	3.8 ± 0.5	3.6 ± 0.5	Flame Photometry

Note: The increase in Na^+^ concentration in the alkaline group originated from the NaHCO_3_/Na_2_CO_3_ buffering system used to stabilize alkalinity.

**Table 2:** Key differentially expressed genes (DEGs) in kidney and liver tissue in response to acute alkaline stress.

Tissue	Gene Symbol	FDR	log2FC	Regulated
Kidney	*acod1*	0.000433265	6.33354	up
	*prlra*	7.10 × 10^−7^	6.290565	up
	*myl13*	0.000314724	5.697281	up
	*klhl38b*	0.000570015	5.502371	up
	*asb10*	0.000205197	5.316526	up
	*slc7a11*	1.18 × 10^−12^	5.309597	up
	*uox*	1.41 × 10^−5^	−7.35524	down
	*fabp10a*	0.000147944	−6.94263	down
	*ucp1*	0.00089816	−6.29334	down
	*c9*	0.001419869	−5.85674	down
Liver	*stard5*	4.39 × 10^−8^	8.261538	up
	*aldh18a1*	1.37 × 10^−6^	8.261132	up
	*higd1a*	3.84 × 10^−32^	6.940015	up
	*egln3*	0.000101412	6.475258	up
	*slc7a11*	3.38 × 10^−43^	5.905609	up
	*angptl3*	1.01 × 10^−32^	−7.33362	down
	*ampd2b*	2.34 × 10^−5^	−6.57406	down
	*rbpjl*	6.77 × 10^−6^	−6.53325	down
	*uox*	1.62 × 10^−5^	−5.90873	down
	*prkcg*	1.70 × 10^−12^	−5.81153	down

## Data Availability

The raw sequencing data for this study have been deposited in the NCBI Sequence Read Archive (SRA) database under accession number PRJNA1346549.

## Supplementary Materials

The following supporting information can be downloaded at: https://www.mdpi.com/article/10.3390/ani15243621/s1. Table S1: Primers used in the study. Table S2: Summary of RNA-Seq data quality control. Figure S1: Sample correlation and clustering analysis. Hierarchical clustering heatmap of all samples based on FPKM values. Figure S2: GO enrichment analysis of differentially expressed genes (DEGs) in liver. Figure S3: KEGG enrichment analysis of differentially expressed genes (DEGs) in liver. Figure S4: GO enrichment analysis of differentially expressed genes (DEGs) in kidney. Figure S5: KEGG enrichment analysis of differentially expressed genes (DEGs) in kidney. Supplementary Material S2: Gene expression data of differentially expressed genes under acute alkaline stress.

## Abbreviations

The following abbreviations are used in this manuscript:

*acod1*	aconitate decarboxylase 1
*aldh18a1*	aldehyde dehydrogenase 18 family member A1
*ampd2b*	adenosine monophosphate deaminase 2b
*angptl3*	angiopoietin like 3
ANOVA	Analysis of Variance
*asb10*	ankyrin repeat and SOCS box containing 10
*atp6v1ab*	ATPase H^+^ Transporting V1 Subunit A, isoform b
BCA	Bicinchoninic acid assay
BH	Benjamini–Hochberg procedure
*c9*	complement C9
CAT	Catalase
cDNA	complementary DNA
CV	central vein
DEGs	Differentially Expressed Genes
*dgat2*	Diacylglycerol O-acyltransferase 2
*egln3*	egl-9 family hypoxia inducible factor 3
*fabp10a*	fatty acid binding protein 10a, liver basic
FDR	False Discovery Rate
FPKM	Fragments Per Kilobase per Million bases
Gb	Gigabases
GC	Guanine-Cytosine content
GF	Gill Filaments
GL	Gill Lamellae
GO	Gene Ontology
GSH	Glutathione
GSH-Px	Glutathione Peroxidase
H&E	Hematoxylin-Eosin
HC	hepatocytes
HIF-1	Hypoxia-Inducible Factor 1
*higd1a*	HIG1 hypoxia inducible domain family member 1A
*hsp90aa1.2*	Heat shock protein 90 alpha family class A member 1.2
HS	hepatic sinusoids
*ldha*	Lactate dehydrogenase A
IF	inflammatory cell infiltration
KEGG	Kyoto Encyclopedia of Genes and Genomes
Kh	karyorrhexis
*klhl38b*	kelch-like family member 38b
LC50	Median lethal concentration
MAS	marker-assisted selection
MDA	Malondialdehyde
MS-222	Tricaine methanesulfonate
*myl13*	myosin, light chain 13
NCBI	National Center for Biotechnology Information
PCO2	Partial pressure of Carbon Dioxide
*pfkfb3*	6-phosphofructo-2-kinase/fructose-2,6-bisphosphatase 3
Pn	pyknosis
PQC	protein quality control
*prkcg*	protein kinase C gamma
*prlra*	prolactin receptor a
PVC	pavement cells
qRT-PCR	quantitative real-time PCR
*rbpjl*	recombination signal binding protein for immunoglobulin kappa J region like
RNA-Seq	RNA Sequencing
ROS	Reactive Oxygen Species
RSEM	RNA-Seq by Expectation-Maximization
SD	standard deviation
*slc7a11*	solute carrier family 7 member 11
SOD	Superoxide Dismutase
SRA	Sequence Read Archive
*stard5*	StAR related lipid transfer domain containing 5
*tcirg1b*	T-cell immune regulator 1, member b
*ucp1*	uncoupling protein 1
*uox*	urate oxidase
VD	vacuolar degeneration
