# Supplementary material for: Transcriptomic and Physiological Responses Reveal a Time-Associated Multi-Organ Injury Pattern in European Perch (Perca fluviatilis) Under Acute Alkaline Stress
Source: Animals (Basel). 2025 Dec 16;15(24):3621. doi: 10.3390/ani15243621 (PMC12729543; doi:10.3390/ani15243621)
Supplement: Supplementary file 1 [file animals-15-03621-s001.zip › Supplementary Material S1.pdf]

Table S1. Primers used in the study

Primer name	Sequence（5′—3′）
β-actin-F	AACCAACGCCCAACAACTTC
β-actin-R	ACGTTCTCCTTCATCGTTCCAG
acod1-F	TGCTGCGCAAGGGTATTACA
acod1-R	GTCCTGGTTCCTAACAGCCC
slc7a11-F	CTGCAGCATTGGCAGTCATC
slc7a11-R	CCGTCCATGTCACGCTCATA
uox-F	AACAGGCTACGGCAAGAACA
uox-R	TTCTTGATGGTGTCGGTGGG
c9-F	AAGCCTTGTCAGAACGGAGG
c9-R	TCGCTCCGCTACATCTTGAC
stard5-F	TTCACCCTTTGTGGGTGCTT
stard5-R	ATACTGGGACTGGCCTTTGC
egln3-F	TCACTCTGGAGGAGTCGAGG
egln3-R	GTTTGAAGCGAGACGAAGGC
angptl3-F	ACAGGGCCCTCATATTCCCT
angptl3-R	AAGCGCTGGTAGGAAGCAAT
prkcg-F	ATGCAGCAGAGATAGCGGTC
prkcg-R	GGGCGATGTAGTCAGGAGTG

Table S2. Summary of RNA-Seq data quality control

SampleID	rawReads	cleanReads	rawBase	cleanBase	Q20	Q30	GC
Control_Kidney_Rep1	50,297,400	50,290,452	7,544,610,000	7,509,973,052	98.8	96.45	50.73
Control_Kidney_Rep2	45,153,258	45,152,158	6,772,988,700	6,744,321,652	98.82	96.44	50.2
Control_Kidney_Rep3	46,045,310	46,039,148	6,906,796,500	6,880,447,888	98.69	96.21	48.38
Control_Liver_Rep1	52,227,836	52,165,640	7,834,175,400	7,787,847,811	98.81	96.5	50.06
Control_Liver_Rep2	48,841,942	48,697,022	7,326,291,300	7,281,089,097	98.2	94.86	49.83
Control_Liver_Rep3	40,681,298	40,631,572	6,102,194,700	6,067,123,528	98.74	96.27	50.21
Exp_Kidney_Rep1	38,340,052	38,306,706	5,751,007,800	5,724,855,259	98.65	96.12	47.61
Exp_Kidney_Rep2	40,490,858	40,466,652	6,073,628,700	6,042,110,530	98.61	95.89	47.77
Exp_Kidney_Rep3	41,939,270	41,900,472	6,290,890,500	6,256,118,364	98.59	95.9	47.88
Exp_Liver_Rep1	36,994,112	36,965,694	5,549,116,800	5,523,561,177	98.78	96.36	49.1
Exp_Liver_Rep2	42,466,978	42,461,214	6,370,046,700	6,345,121,686	98.8	96.37	48.82
Exp_Liver_Rep3	39,014,240	38,980,574	5,852,136,000	5,822,578,645	98.78	96.36	48.89

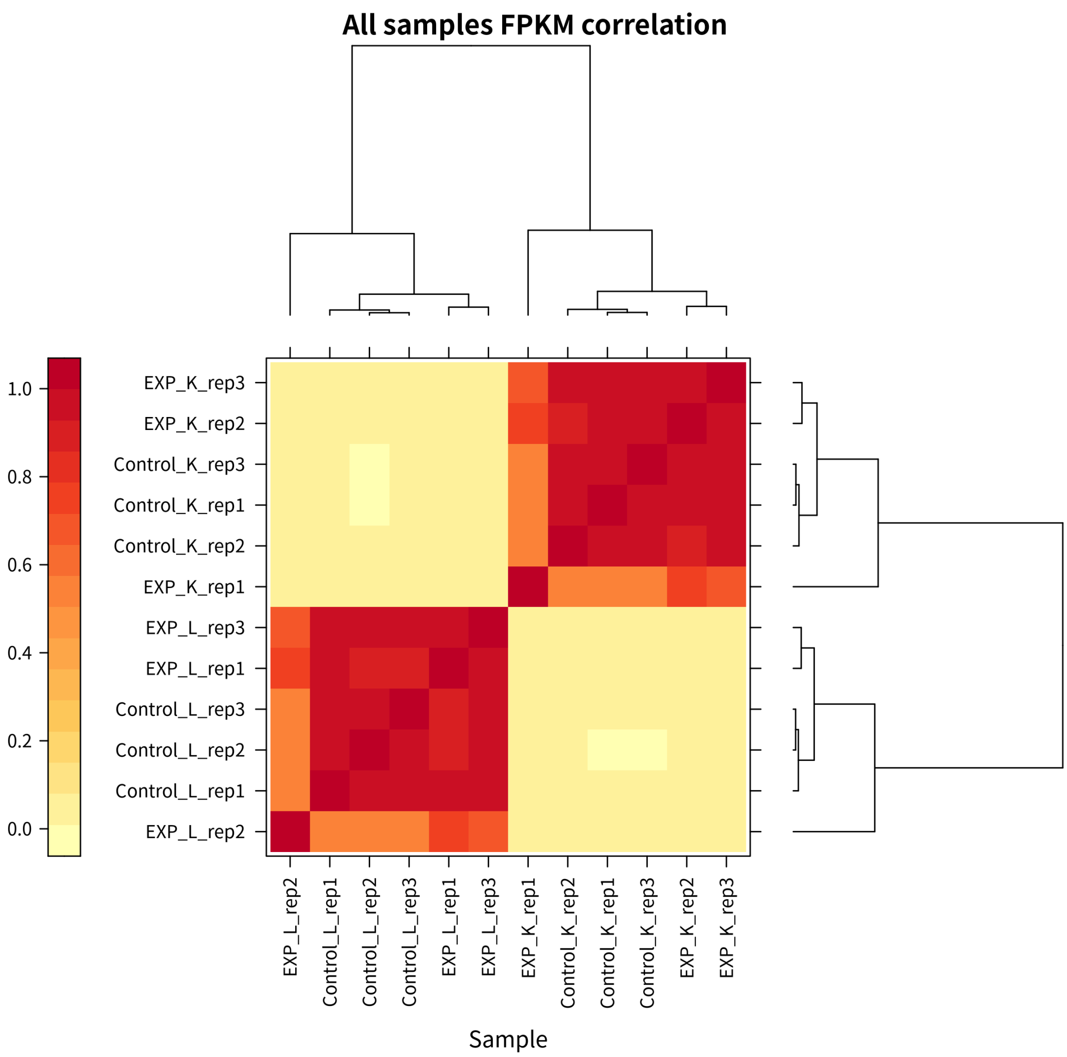

Figure S1. Sample correlation and clustering analysis. Hierarchical clustering heatmap of all samples based on FPKM values. The color scale indicates the Pearson correlation coefficient. Samples cluster primarily by tissue (Kidney vs. Liver) and treatment (Control vs. Experiment).

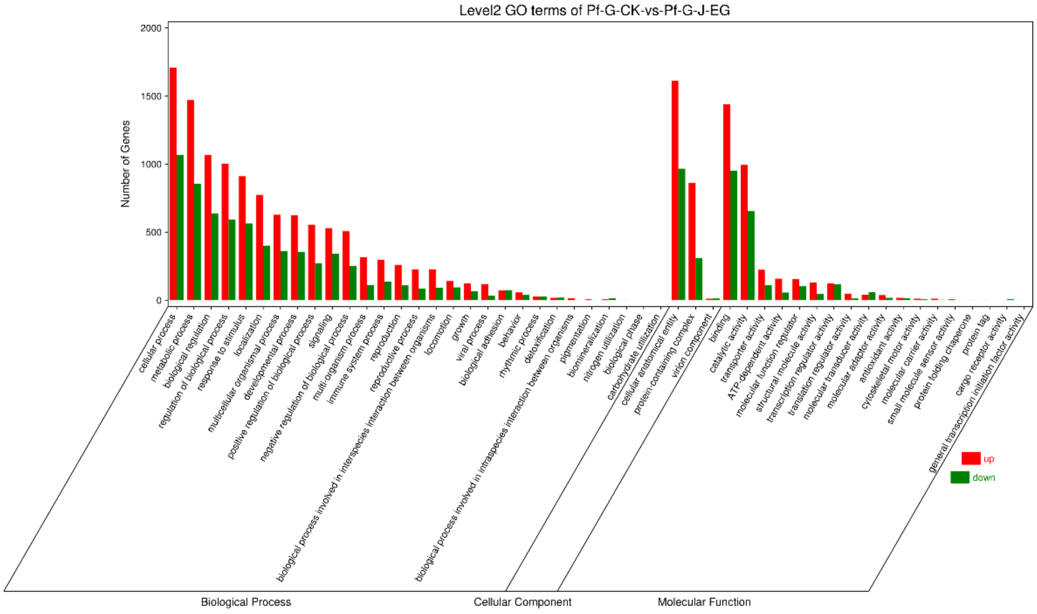

Figure S2. GO enrichment analysis of differentially expressed genes (DEGs) in liver. The most significantly enriched GO terms in biological process (BP), cellular component (CC), and molecular function (MF) categories.

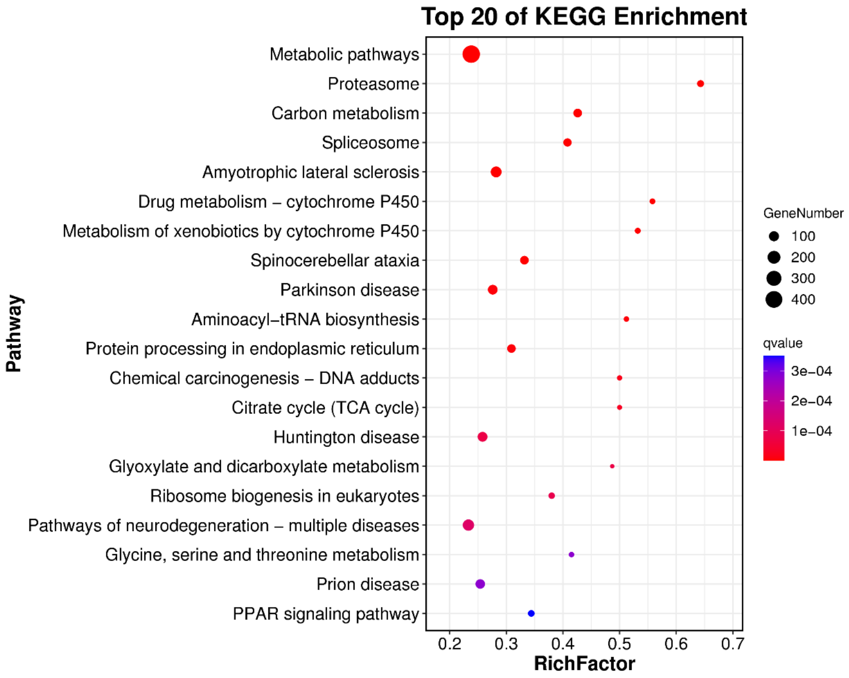

Figure S3. KEGG enrichment analysis of differentially expressed genes (DEGs) in liver. The vertical axis represents the name of the term or pathway, and the horizontal axis represents the rich factor. The size of the dot indicates the number of DEGs enriched in that term, and the color represents the range of the false discovery rate (FDR) corrected P-value (Q-value).

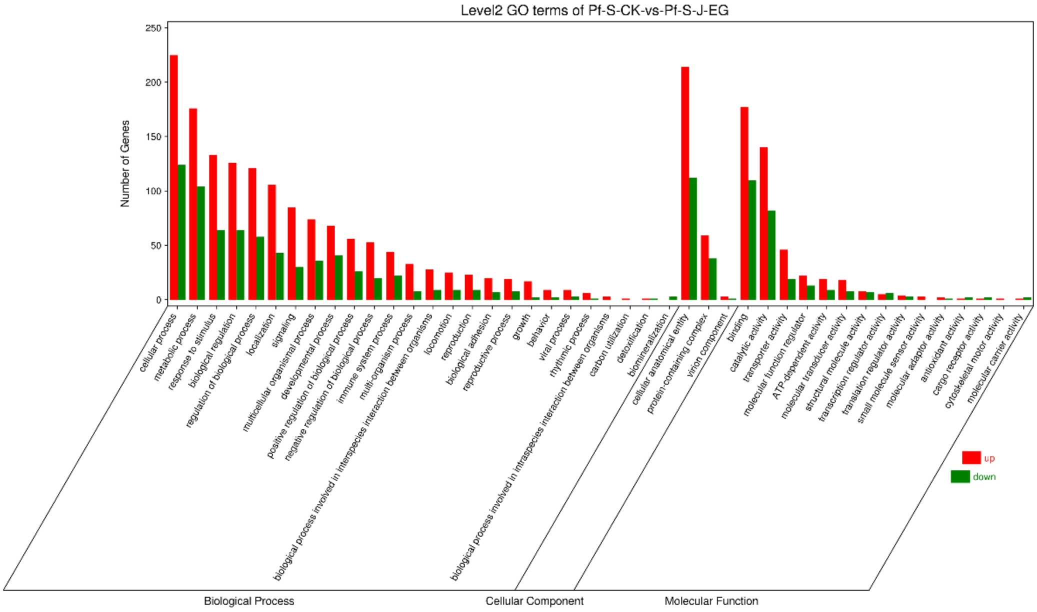

Figure S4. GO enrichment analysis of differentially expressed genes (DEGs) in kidney. The most significantly enriched GO terms in biological process (BP), cellular component (CC), and molecular function (MF) categories.

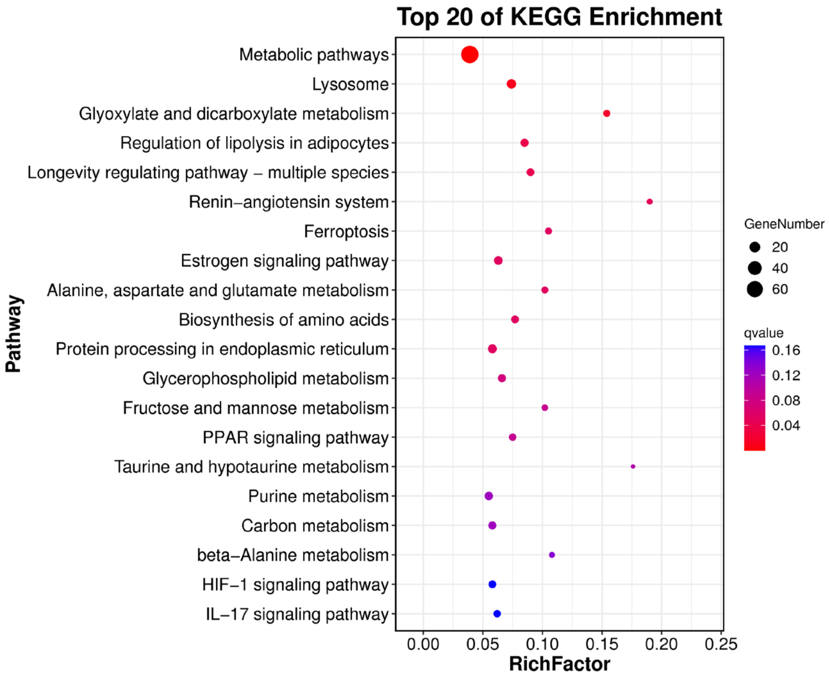

Figure S5. KEGG enrichment analysis of differentially expressed genes (DEGs) in kidney. The vertical axis represents the name of the term or pathway, and the horizontal axis represents the rich factor. The size of the dot indicates the number of DEGs enriched in that term, and the color represents the range of the false discovery rate (FDR) corrected P-value (Q-value).
